# Changes in Eukaryotic Phytoplankton Community Structure Induced by a Typhoon Event: A Case Study in Zhanjiang Bay, China

**DOI:** 10.3390/microorganisms13112609

**Published:** 2025-11-16

**Authors:** Hui Huang, Junze Wu, Zhangxi Hu, Fuyuan Zeng, Menghan Gao, Yu Luo, Shafira Citra Desrika Putri, Yulei Zhang

**Affiliations:** 1 Guangdong Provincial Key Laboratory of Aquatic Animal Disease Control and Healthy Culture, Key Laboratory of Marine Ecology and Aquaculture Environment of Zhanjiang, College of Fisheries, Guangdong Ocean University, Zhanjiang 524088, China; 13544539860@stu.gdou.edu.cn (H.H.); 13418580541@163.com (J.W.); huzx@gdou.edu.cn (Z.H.); cengfuyuan1@stu.gdou.edu.cn (F.Z.); gaomenghan0914@163.com (M.G.); luoyu526gdhy@163.com (Y.L.); shaci13@stu.gdou.edu.cn (S.C.D.P.); 2 Guangdong Laboratory of Marine Ecology Environment Monitoring and Warning, Guangdong Ocean University, Zhanjiang 524088, China

**Keywords:** metabarcoding, phytoplankton, typhoon, disturbance response, Zhanjiang Bay

## Abstract

The Zhanjiang Bay ecosystem, frequently influenced by typhoons, represents a highly dynamic coastal environment where elucidating phytoplankton responses to extreme disturbances is essential for sustainable management. This study investigated the impacts of Typhoon Prapiroon on phytoplankton community composition and distribution by employing high-throughput sequencing of the 28S rDNA D1–D2 regions. A total of 137 species belonging to 46 genera was identified, with the ten dominant genera collectively contributing more than 85% of the total abundance, exhibiting substantial shifts in community structure following the typhoon. Salinity was identified as the predominant environmental driver shaping phytoplankton distribution, while temporal analyses revealed lagged community responses to post-typhoon conditions. Moreover, biotic interactions among taxa further influenced patterns of community restructuring. These findings enhance the understanding of phytoplankton resilience mechanisms under extreme climatic disturbances. The integration of phytoplankton monitoring into coastal early warning systems is recommended to inform adaptive management strategies and mitigate ecological risks associated with the increasing frequency and intensity of typhoons, thereby supporting the sustainable use and conservation of estuarine and coastal ecosystems.

## 1. Introduction

Typhoons are intense cyclonic systems that occur in tropical and subtropical oceans, which are among the most powerful meteorological phenomena on Earth [1]. As a typical extreme weather event, typhoons are often accompanied by heavy rainfalls, strong winds, and storm surges, causing severe impacts on natural ecosystems and human social activities [2]. In recent years, influenced by global climate warming, the frequency and intensity of typhoons have been increasing, and their ecological impacts have garnered increasing attention [3,4,5]. Globally, the South China Sea is one of the most active regions for typhoon activity [6]. During the passage of a typhoon, intense hydrodynamic processes can lead to significant changes in environmental factors, such as sea temperatures, salinity, and nutrient concentration. This phenomenon is especially true in nearshore areas significantly influenced by land-derived runoff, where the sudden disturbances caused by typhoons are likely to disrupt the existing ecological balance. However, the succession trends of phytoplankton communities, the competitive dynamics of dominant species, and their dynamic responses to environmental factors under typhoon disturbances still require systematic analyses.

Algal blooms, an ecological phenomenon characterized by excessive phytoplankton proliferation, have frequently occurred in coastal areas worldwide in recent years and are becoming one of the major environmental issues faced by marine ecosystems [7]. Algal blooms disrupt phytoplankton community structure and function, and may even potentially release toxins and cause hypoxia, resulting in significant mortality of fishery resources [8]. This poses a serious threat to the fishery economy, ecosystem, and even public health. Studies have shown that the occurrence of algal blooms is driven by the synergistic effects of multiple environmental factors [9]. Among these, typhoons, as significant external disturbances to nutrient and physicochemical factors, have attracted widespread attention due to their close association with algal blooms. Typhoon passages have also been frequently associated with algal bloom events around the world [10], suggesting that typhoons may be a key triggering mechanism for algal blooms. Phytoplankton serve as important biological indicators for water quality monitoring and constitute a major source of marine primary productivity, contributing approximately 50% of global net primary production, while also acting as key components of biological and inorganic carbon pools and playing a central role in the global carbon cycle through the “biological pump” mechanism [11]. Given the fundamental role of phytoplankton in marine ecosystems, studying their community structures and dynamics is of significant importance for understanding the ecological processes and response mechanisms to environmental changes.

Zhanjiang Bay, as a typical semi-enclosed bay, has a relatively weak water exchange capacity. Combined with high-intensity human activities such as aquaculture, industry, and tourism, as well as the input of land-derived pollutants carried by the Zhujiang River runoff, the bay has been in a state of long-term eutrophication, leading to frequent algal bloom events [12]. Frequent typhoon disturbances, combined with local eutrophication, further exacerbate the risk of algal bloom outbreaks. In addition, as an important aquaculture hub in China [13] and a major distribution area for mangroves in South China [14], the ecological stability of Zhanjiang Bay is closely linked to the structure of phytoplankton communities within the bay.

Zhanjiang Bay is frequently affected by typhoons, with an average of up to ten typhoons directly or indirectly impacting the region each year [15]. Due to these frequent disturbances and the inherent vulnerability of coastal ecosystems [16], this area serves as a representative area for investigating the relationship between typhoons and phytoplankton communities.

This study aimed to elucidate the responses of eukaryotic phytoplankton and environmental factors following a typhoon using metabarcoding technology, and to illustrate the ecological resilience of these communities under extreme climatic events. It also sought to identify the key environmental drivers of community succession and to reveal the intricate species-species and species-environment relationships, providing a finer-scale and multidimensional perspective on community dynamics under extreme disturbances. To achieve this, five sampling stations were established in Zhanjiang Bay during the passage of Typhoon Prapiroon in 2024, covering both surface and bottom water layers across four temporal stages. The insights gained from this research will not only assist coastal managers in formulating adaptive strategies to protect ecosystems and mitigate disaster impacts but also provide a scientific basis for improving early warning systems, optimizing resource allocation, and supporting evidence-based policy decisions to enhance the resilience and sustainability of coastal regions.

## 2. Materials and Methods

### 2.1. Study Area and Typhoon Prapiroon Description

**Figure 1:**
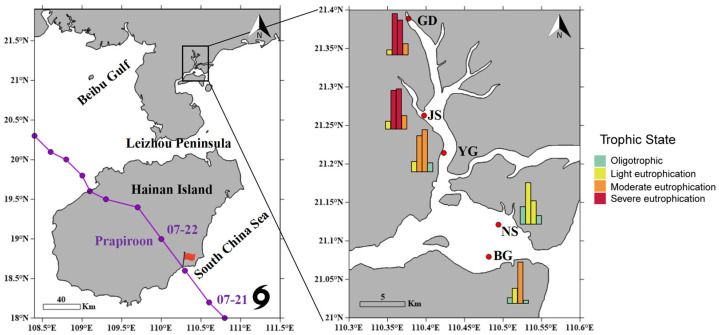
The path of Typhoon Prapiroon and the location of sampling sites in Zhanjiang Bay, China. Temporal trends in eutrophication levels at different sites and stages. The bars in the histogram represent the eutrophication level at different stages for each sampling station in sequence. An E value less than 1 indicates oligotrophic conditions, while E ≥ 1 indicates eutrophication. Specifically, 1 ≤ E ≤ 3 represents mild eutrophication, 3 < E ≤ 9 represents moderate eutrophication, and E > 9 represents severe eutrophication.

Zhanjiang Bay, a semi-enclosed bay, is located on the northeastern side of the Leizhou Peninsula (Figure 1), adjacent to the South China Sea. Nutrient inputs primarily originate from the Suixi River and the western Guangdong coastal current. Due to poor water exchange capacity, the bay is prone to eutrophication. The eutrophic status was determined based on the eutrophication index (E), calculated according to the formula and evaluation criteria outlined in the China Marine Ecological and Environmental Status Bulletin (https://www.mee.gov.cn/hjzl/sthjzk/jagb/) (accessed on 1 September 2024).

The trajectory of Typhoon Prapiroon was obtained from the China Meteorological Administration (https://typhoon.weather.com.cn/gis/typhoon_m.shtml) (accessed on 1 September 2024). Typhoon Prapiroon formed over the northwestern Pacific Ocean on the afternoon of 15 July 2024. At 1:00 a.m. on 22 July, it made landfall in Hainan Province as a tropical storm, with maximum sustained winds near the center reaching 28 m·s^−1^. Influenced by Typhoon Prapiroon, monitoring data from the Guangdong Provincial Hydrology Bureau (http://slt.gd.gov.cn/zsdw8721/content/mpost_4464609.html) (accessed on 1 September 2024) indicated that Zhanjiang recorded an average rainfall of 52.7 mm on 22 July 2024. Several terrestrial runoff channels experienced rising water levels, and storm surges of 40–100 cm were observed along the eastern coast of Zhanjiang, China. The Zhanjiang Meteorological Bureau recorded a maximum gust of 30.5 m·s^−1^ (http://gd.cma.gov.cn/zjsqxj/) (accessed on 1 September 2024).

### 2.2. Sample Collection, DNA Extraction, PCR Amplification, and Sequencing

The typhoon process was divided into different stages. Four sampling stages were defined: Stage 0 (20 July, before landfall), Stage 1 (25 July, 3 days after landfall), Stage 2 (1 August, 10 days after landfall), and Stage 3 (7 August, 15 days after landfall). The field investigation dates corresponded to the different stages. Triplicate surface (0.5 m) and bottom water samples (about 1 m above the bottom) were collected from five sampling sites using an acrylic water sampler (LB-8500, Qingdao Loobo Environmental Protection Group Co., Ltd., Qingdao, China) (Figure 1), and filtered through 0.25 μm polycarbonate membranes (Pall Corporation, Ann Arbor, MI, USA). A total of 120 water samples were prepared and stored at −80 °C for short-term preservation before further analysis.

Total genomic DNA was extracted using the DNeasy PowerWater Kit (Qiagen, Hilden, Germany) according to the manufacturer’s protocols. The D1–D2 regions of the 28S rDNA gene were amplified by PCR using the primers LSU335F (5′-ACCGATAGCA(G)AACAAGTA-3′) and LSU714R (5′-TCCTTGGTCCGTGTTTCA-3′) with PCR amplification and sequencing were performed as previously described [17]. PCR products were separated on 2% agarose gels and purified using the AxyPrep DNA Gel Extraction Kit (Axygen Biosciences, Union City, CA, USA) following the manufacturer’s instructions. Purified PCR products were quantified using Qubit^®^3.0 (Life Invitrogen, Carlsbad, CA, USA), and every twenty-four amplicons with different barcodes were mixed equally. The pooled DNA product was used to construct the Illumina Pair-End library following Illumina’s genomic DNA library preparation procedure. Then, the amplicon library was paired-end sequenced (2 × 300) on an NGS platform (Shanghai BIOZERON Biotech. Co., Ltd., Shanghai, China) according to standard protocols.

Raw FASTQ files were first demultiplexed using Trimmomatic [18] (http://www.usadellab.org/cms/) (accessed on 1 September 2024) and in-house Perl scripts according to the barcode sequences information for each sample with the following criteria: (i) The 300 bp reads were truncated at any site receiving an average quality score < 20 over a 10 bp sliding window, discarding the truncated reads that were shorter than 50 bp. (ii) Exact barcode matching, up to two nucleotide mismatches in primer matching, or containing ambiguous characters were removed. (iii) Only sequences that overlap longer than 10 bp were assembled according to their overlap sequence. Reads which could not be assembled were discarded.

The passed sequences were dereplicated and processed using the DADA2 algorithm (recommended by QIIME 2) to identify insertion-deletion (indel) mutations and substitutions [19]. Paired-end reads were trimmed and filtered with a maximum expected error of 2 per read (maxEE = 2). After merging paired reads and removing chimeras, each 28S rDNA sequence (here referred to as ASVs) was compared against the Silva LSURef NR99 138.1 28S rDNA database (https://www.arb-silva.de) (accessed on 1 September 2024) using a confidence threshold of 80%.

### 2.3. Analyses of Water Chemical Indicators

At each station, temperature, salinity, dissolved oxygen (DO), pH, and water depth were measured using a handheld multi-parameter instrument (LH-T600, Zhejiang Lohand Environment Technology Co., Ltd., Hangzhou, China) and depth sounder (SM-5A, Speedtech, St. George, UT, USA). The concentrations of SiO_3_^2−^, PO_4_^3−^, NO_2_^−^, NO_3_^−^, and NH_4_^+^ were measured using an automated nutrient analyzer (SmartChem 200, AMS, Milano, Italy). Chlorophyll *a* concentration and chemical oxygen demand (COD) were determined using the ethanol extraction method and the alkaline potassium permanganate method, respectively. The analytical procedures followed the Specifications for Oceanographic Monitoring—Part 4: Seawater Analysis (GB 17378.4-2007) [20], a national standard issued in China.

### 2.4. Statistical Analyses

Statistical analyses and data visualizations were conducted using RStudio (version 4.3.2). Map visualizations were generated using Surfer. To determine the significant differences in environmental variables across different stages of the typhoon event, we conducted pairwise PERMANOVA analyses based on the overall multivariate analysis of variance (MANOVA). The Benjamini–Hochberg method was applied to correct for multiple hypothesis testing corrections and enhance the robustness of the analysis. Using the R package vegan (version 2.7.1) to perform beta diversity analysis, principal component analysis (PCA), principal coordinate analysis (PCoA), and non-metric multidimensional scaling (NMDS). Spearman’s rank correlation was used to analyze the relationship between phytoplankton community composition and environmental factors across different stages of development. Network analysis tools included the R packages psych (version 2.5.3) and corrplot (version 0.95), with visualization performed using ggraph (version 2.2.1). To visually illustrate the relationship between different groups and site distributions, the circlize package (version 0.4.16) was used for visualization. Non-linear least squares fitting of neutral model parameters was performed using the minpack.lm package (version 1.2.4), followed by visualization of the fitting results using the grid package (version 4.3.2). To identify key variables driving changes in phytoplankton community composition during the typhoon impact, a random forest model was constructed using the randomForest package (version 4.7-1.2), with results visualized through heatmaps generated by the pheatmap package (version 1.0.13). Pie charts, bar plots, and line graphs were created using the R package ggplot2 (version 3.5.2).

### 2.5. Random Forest Analysis

#### 2.5.1. Model Development

Random Forest (RF) is an ensemble learning method based on decision trees, where a large number of weak learners are integrated to improve overall predictive performance. We used the createDataPartition function to split the complete dataset (*n* = 6960) into training and testing sets in a 7:3 ratio, with a fixed random seed for training. The model construction process is divided into two stages: calibration and validation. In the calibration stage, multiple gradient steps (ranging from 100 to 2000) are used to build various models to determine the optimal number of trees (ntree) and to optimize the number of features considered for each node split (mtry). After determining the values for ntree and mtry, the tree structure parameters, including the minimum terminal node size (nodesize) and the maximum number of nodes (maxnodes), are further adjusted. In the validation stage, 10-fold cross-validation (10-fold CV) is used to assess the stability and generalizability of the model (Figure S1). The normalized root mean square error (NRMSE) for each fold is recorded, and error fluctuations are compared through visualization [21]. Based on the cross-validation evaluation results, the model is iteratively trained, and the parameter configuration with the best predictive performance is selected. The final evaluation was conducted on the test set to assess the model’s ability to capture complex non-linear relationships and its generalization performance.

#### 2.5.2. Inter-Species Relationships and Local Effects Analyses

The potential ecological relationships between species were analyzed using RF. The abundance of the target species was set as the response variable, while the abundance of other species served as predictor variables. Models were constructed separately for each target species using the randomForest function. After the model was constructed, the test set (30% of the data) was used for prediction and evaluation. The correlation coefficient (R^2^), root mean square error (RMSE), and normalized root mean square error (NRMSE) were employed to assess the predictive performance and goodness of fit of the model [22]. Based on the trained random forest model, the iml package was used to calculate and visualize the marginal effects of selected variables on the abundance prediction of the response variable.

#### 2.5.3. Variable Importance and Correlation

To assess the relative importance of environmental factors in predicting phytoplankton abundance, we used the percentage increase in mean squared error (%IncMSE), a variable importance metric from the RF model, as it better reflects their impact on prediction error for continuous variables [23]. To identify the main environmental drivers of each species’ abundance, separate RF models were built and evaluated with rfPermute (99 permutations). Variables significant at *p* < 0.05 were retained; their percentage increase in mean squared error (IncMSE) indicates the loss in predictive power when permuted, and the R^2^ of the final tree measures model fit. Spearman correlations were then computed between each significant variable and species abundance to visualize the direction and strength of the relationships.

## 3. Results

### 3.1. Composition and Relative Abundance of Phytoplankton Communities in Zhanjiang Bay During Typhoon Prapiroon

**Figure 2:**
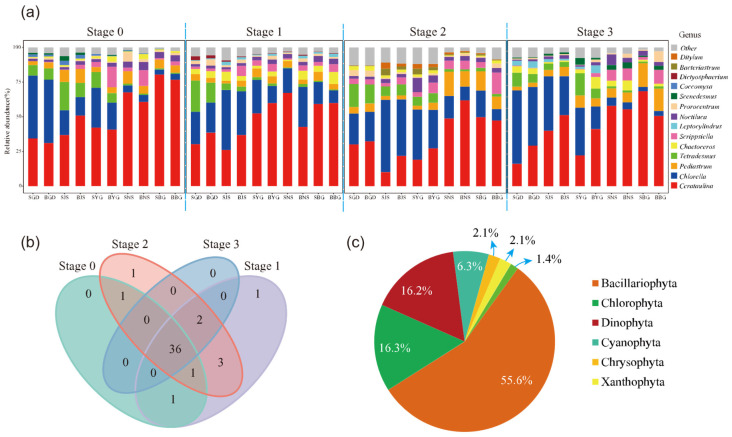
Analysis of algal community diversity, (**a**) temporal changes in genus-level composition, “S” and “B” represent surface and bottom water samples, respectively; the following two letters indicate the sampling station codes. (**b**) Venn diagram of species composition at different stages and (**c**) total species abundance by phylum.

We obtained 358,594 raw 28S rDNA sequence reads from 120 samples across four time points, and 236,533 ASVs annotated as phytoplankton after processing the Illumina sequencing results using DADA2. Shannon–Wiener curve analysis indicated that the sequencing depth had reached saturation, suggesting that the sequencing data were sufficient and large enough to reflect the majority of microbial species information in the samples (Figure S2). After averaging the three parallel samples from each site, the composition and relative abundance of phytoplankton at the genus level were compared across different sites and time stages. The species composition diagram displays the top 10 taxa in each site, with the remaining grouped as “Other” (Figure 2a).

The dominant phytoplankton groups were identified as *Cerataulina* and *Chlorella* (Figure 2a). Among the 40 sample groups (averaged from parallel samples), 30 were dominated by *Cerataulina* and 10 by *Chlorella*. The highest relative abundance of *Cerataulina* was 80.5% (stage 0, BG), with an average ASV abundance of 52.15%, while *Chlorella* reached a maximum of 52.9% (stage 3, GD), with an average ASV abundance of 22.51%. Eight genera consistently ranked among the top ten in relative abundance across all four stages: *Cerataulina*, *Chlorella*, *Tetradesmus*, *Pediastrum*, *Chaetoceros*, *Scrippsiella*, *Noctiluca*, and *Prorocentrum*. One unique genus was identified at the sampling sites during each of Stage 1 and Stage 2 (Figure 2b): *Euglena* (Stage 1) and *Alexandrium* (Stage 2).

All 692,733 quality-filtered eukaryotic phytoplankton sequences were further classified into different taxonomic levels, from kingdom to species, resulting in a total of 6 phyla, 11 classes, 28 orders, 37 families, 46 genera, and 137 species. Among them, Bacillariophyta had the highest average relative abundance (60.6%), followed by Chlorophyta (20.4%), Dinophyta (11.7%), Chrysophyta (4.4%), Xanthophyta (1.5%), and Euglenophyta (0.7%) (Figure 2c).

### 3.2. Impacts of Typhoon Prapiroon on Environmental Conditions and Phytoplankton Community Structure

**Figure 3:**
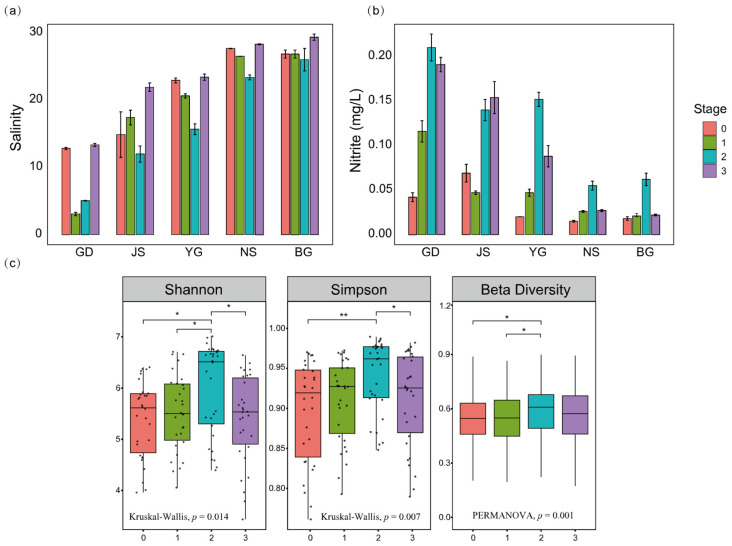
Analysis of changes in environmental parameters and community diversity across different stages. (**a**) Salinity variation trends across sampling sites. (**b**) Nitrite variation trends across sampling sites. (**c**) Stage-wise differences in the Shannon–Wiener index, Simpson index, and beta diversity of algal communities. Shannon and Simpson diversity indices were evaluated using the Kruskal–Wallis test, followed by Dunn’s post hoc test with Benjamini–Hochberg (BH) correction for multiple comparisons. Beta diversity differences among and between stages were assessed using PERMANOVA with 999 permutations. (* *p* < 0.05, ** *p* < 0.01; BH-adjusted).

The analyses of environmental variables at different stages, both before and after the typhoon, revealed significant differences among the stages (MANOVA, *p* < 0.05). Further pairwise comparisons revealed significant differences between pre-typhoon and 10 days post-typhoon and between pre-typhoon and 14 days post-typhoon (PERMANOVA, adjusted *p* < 0.05, BH). Compared to the other stages, the second stage had the lowest mean values of salinity, temperature, DO, and pH, while the average concentrations of nitrite and silicate were higher than in the other stages (Figure 3a,b and Figure S3). The problem of eutrophication is also primarily concentrated in the first and second stages after the typhoon (Figure 1). Additionally, these findings together indicate a land-to-sea gradient, with nutrient concentrations and eutrophication levels decreasing from estuarine areas to offshore areas.

The impact of Typhoon Prapiroon on the phytoplankton community diversity in Zhanjiang Bay is significant, with different evaluation indices showing a consistent trend (Figure 3c). The diversity of the algal community showed an increasing trend after the passage of the typhoon, reaching its peak during the second stage, ten days after the typhoon. However, a significant decline was observed in the third stage, fourteen days after the typhoon, compared to the second stage. Overall, the diversity of the algal community was higher after the typhoon passage than before, with the most significant increase observed in the second stage.

In summary, the environmental variables and community diversity indices during the second stage following the typhoon’s passage collectively highlight the uniqueness and representativeness of this stage. The high diversity observed during this stage provides a crucial entry point for subsequent research, particularly in exploring the relationship between environmental changes and community dynamics, which warrants further in-depth investigation.

### 3.3. Spatiotemporal Distribution Patterns of Algal Communities During Typhoon Prapiroon

**Figure 4:**
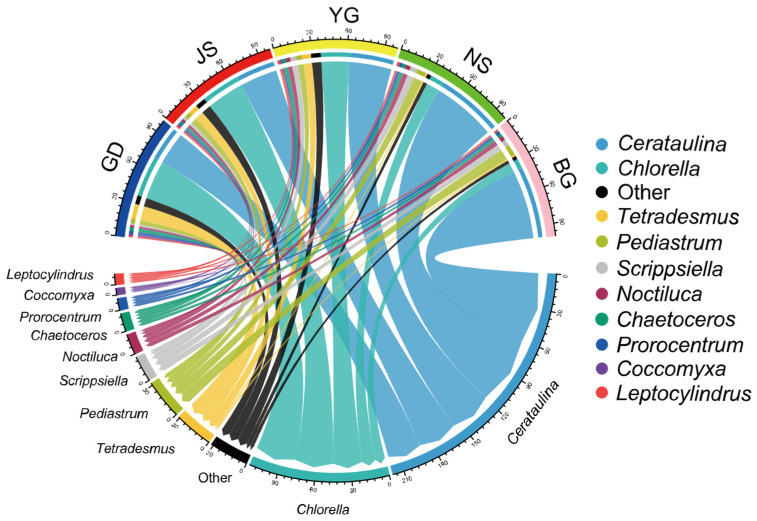
Relationship between the top ten species at the genus level in terms of abundance among the 120 samples and different sampling sites.

Phytoplankton in different taxonomic groups exhibited distinct spatial distribution patterns across Zhanjiang Bay. Among the top ten most abundant genera (accounting for over 85% of all samples), *Cerataulina*, *Scrippsiella*, and *Noctiluca* showed a gradient increase from north to south in the bay, while *Chlorella*, *Tetradesmus*, and *Coccomyxa* displayed the opposite trend (Figure 4). Species outside the top ten in abundance were primarily distributed at nearshore stations in the northern part of Zhanjiang Bay (GD, JS, YG), where salinity levels were all below 25. As salinity varied, a gradual succession in dominance between *Cerataulina* and *Chlorella* was also observed.

**Figure 5:**
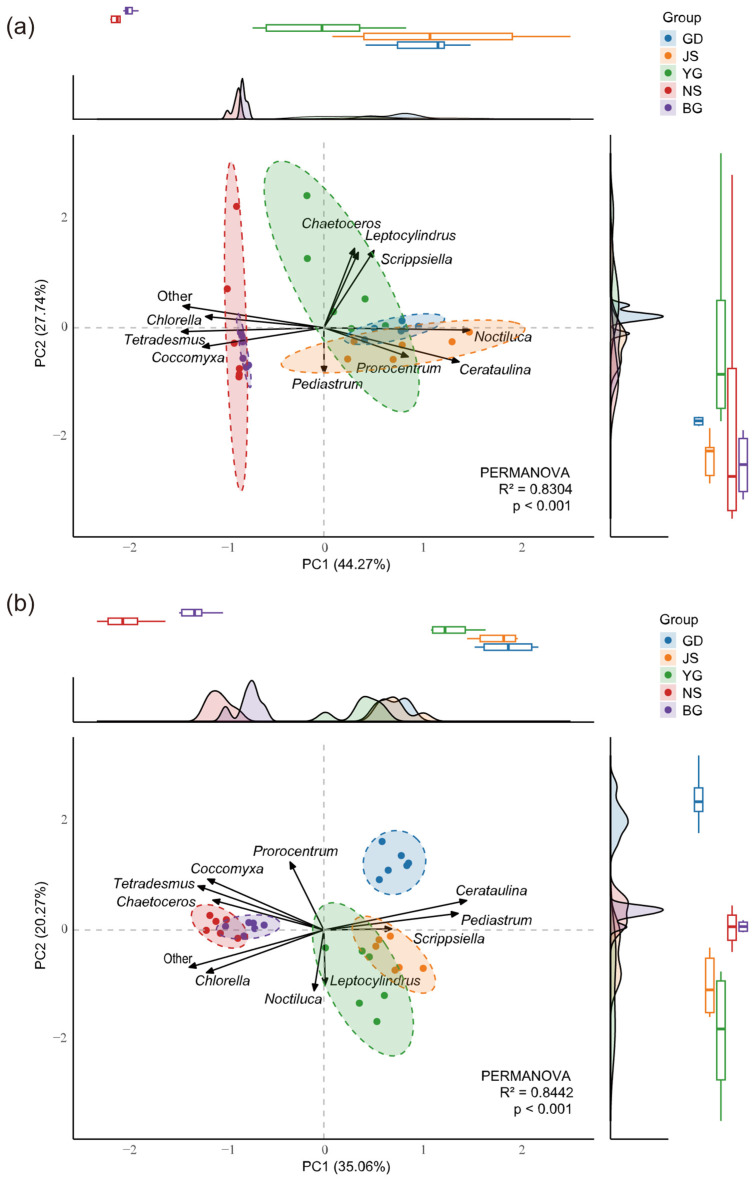
Principal Component Analysis (PCA) of Stage 0 before typhoon (**a**) and Stage 2 after typhoon (**b**). Differences in community abundance among stations were evaluated using PERMANOVA based on Bray–Curtis distances. The axes PC1 and PC2 represent the two dimensions explaining the highest variance. Dashed lines indicate within-group clustering at a 95% confidence interval. Loading vectors represent the contributions of the top 10 most abundant species to the principal components. Marginal density curves and boxplots illustrate the distribution trends and central tendencies of samples along the principal component axes. R^2^ indicates the proportion of community variation explained by grouping.

The distribution patterns of algal communities exhibited spatiotemporal heterogeneity across different stages of the typhoon event. Before the typhoon (Figure 5a), the community structure showed significant geographic variation (PERMANOVA, R^2^ = 0.8304, *p* < 0.01). PCA that after the typhoon, algal abundance across different stations showed a more pronounced separation in principal component space compared to before the typhoon (PERMANOVA, R^2^ = 0.8442, *p* < 0.01) (Figure 5b). Further analysis revealed that the two high-salinity estuarine stations (salinity > 25), NS and BG, exhibited similar trends and concentration in their marginal density curves and boxplots along the principal component axes during these two stages, indicating that their community composition characteristics were relatively similar. In addition, our results revealed that *Chlorella*, *Coccomyxa*, and other algae showed arrow directions opposite to those of *Cerataulina* and *Scrippsiella*, suggesting that they may exhibit a negative ecological correlation in terms of niche differentiation.

**Figure 6:**
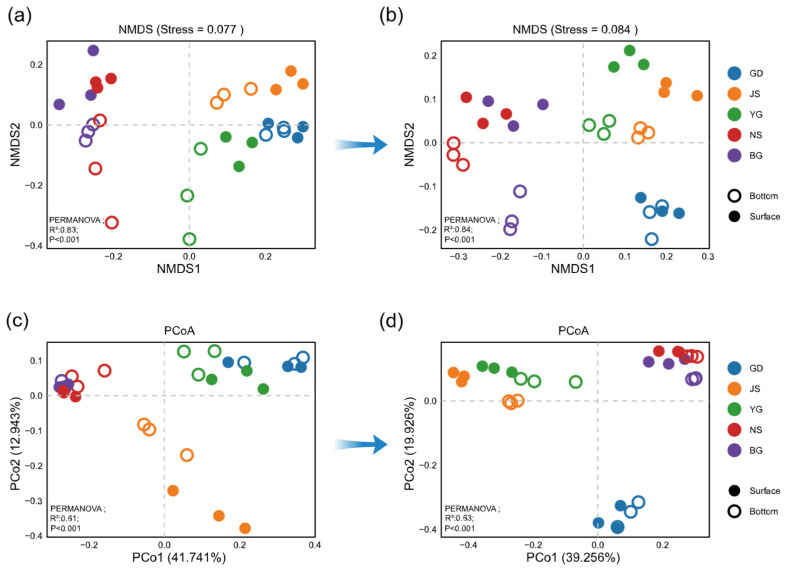
Inter-station differences across stages were analyzed using multiple methods. Non-metric multidimensional scaling (NMDS) plots based on relative abundance were used to visualize differences among stations at Stage 0 (**a**) and Stage 2 (**b**). Principal coordinates analysis (PCoA) plots based on Bray–Curtis distances were used to evaluate beta diversity among stations at Stage 0 (**c**) and Stage 2 (**d**). Beta diversity differences among stations and between water layers were statistically tested using PERMANOVA.

Non-metric multidimensional scaling (NMDS) revealed that phytoplankton communities in different water layers were similar in abundance before (Figure 6a, PERMANOVA, *p* = 0.477) and after the typhoon (Figure 6b, PERMANOVA, *p* = 0.183). Likewise, Beta diversity showed no significant difference between water layers either before (Figure 6c, PERMANOVA, *p* = 0.312) or after the typhoon (Figure 6d, PERMANOVA, *p* = 0.201). Meanwhile, the PCoA results revealed a more distinct separation in phytoplankton community composition among stations after the typhoon compared to the pre-typhoon period (Figure 6c,d). In summary, the differences in phytoplankton abundance and Beta diversity collectively indicate that typhoon disturbance enhanced the spatial heterogeneity of community structure, exacerbating the pre-existing geographical distribution differences. Notably, a clear boundary of community differentiation was observed between the inner bay estuaries (GD, JS, YG) and the outer bay estuaries (NS, BG).

### 3.4. Correlation Analysis Between Phytoplankton Communities and Environmental Factors at Different Stages

**Figure 7:**
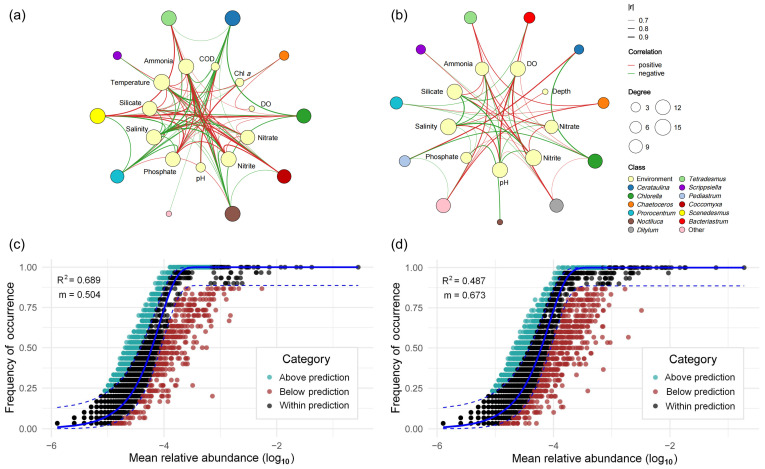
Changes in phytoplankton–environment relationships and community assembly dynamics across typhoon events. (**a**) Association network between the top 10 phytoplankton genera and environmental factors before the typhoon. (**b**) Association network between the top 10 phytoplankton genera and environmental factors during the second stage after the typhoon. Edges: Significant correlations between two variables (Spearman; |r| > 0.6; *p* < 0.05). The neutral community model (NCM) was applied to investigate the assembly of the phytoplankton community. Panels (**c**) and (**d**) represent the periods before the typhoon and the second stage after the typhoon, respectively. The *x*-axis represents the mean relative abundance of each ASV (log_10_-transformed), and the *y*-axis indicates the frequency of occurrence across samples. The occurrences of ASVs above, below, and within the prediction are shown in turquoise, red, and dark dots.

To investigate species–species and species–environment interactions within the phytoplankton community of Zhanjiang Bay, correlations between the top ten genera and environmental factors were analyzed. The beta diversity and network analyses provided complementary insights: beta diversity revealed the distinct community composition of the second stage at a broader scale, whereas the network analysis captured finer species-environment associations and dynamic interaction patterns, offering a more comprehensive understanding of temporal community changes. The results showed that after the typhoon, new nodes representing *Bacteriastrum*, *Ditylum*, and *Pediastrum* were added to the network. In contrast, the node representing temperature disappeared (Figure 7b). In terms of the changes in connectivity, after the typhoon, *Cerataulina* (−11), *Chlorella* (−5), *Prorocentrum* (−5), Other (+5), Silicate (−5), Nitrite (−5), Ammonium (−8), Nitrate (−6), and Phosphate (−9) exhibited a reduction in the number of connections, indicating that the association between most nutrient factors and the phytoplankton community weakened. The response intensity of phytoplankton to nutrient changes decreased. After the typhoon, the ecological niche of the dominant species, *Cerataulina*, fluctuated significantly, while the activity of some previously less abundant algal species increased, indicating a shift in community structure. Overall, the network nodes were simplified after the typhoon, reflecting a decrease in community structure stability and signaling that the algal ecosystem had entered a period of reconstruction.

In terms of significantly correlated edges, there were eight significant positive correlations and ten negative correlations between phytoplankton groups before the typhoon (Figure 7a). After the typhoon, there were nine positive correlations and seven negative correlations, indicating that the original competitive or resource differentiation relationships between groups were disrupted and weakened. The number of significant edges between phytoplankton and environmental factors also decreased from 74 before the typhoon (41 positive, 33 negative) to 48 after the typhoon (15 positive, 14 negative), indicating a weakened and delayed overall response of phytoplankton to environmental factors.

The Neutral Community Model (NCM) demonstrated a moderate level of explanatory power in both stages (Figure 7c,d). However, the observed deviations from the model predictions imply that community assembly was not entirely governed by neutral processes. Additional deterministic factors, such as environmental filtering and biotic interactions, may have also contributed to shaping the community structure. Before the typhoon, the model yielded a coefficient of determination (R^2^) of 0.689 and a migration rate (m) of 0.504; both values declined to 0.673 and 0.487, respectively, after the typhoon (Figure 7c,d), suggesting that the influence of non-neutral processes on community assembly increased under disturbance.

### 3.5. Phytoplankton Interactions and Key Environmental Drivers During Typhoon Prapiroon

**Figure 8:**
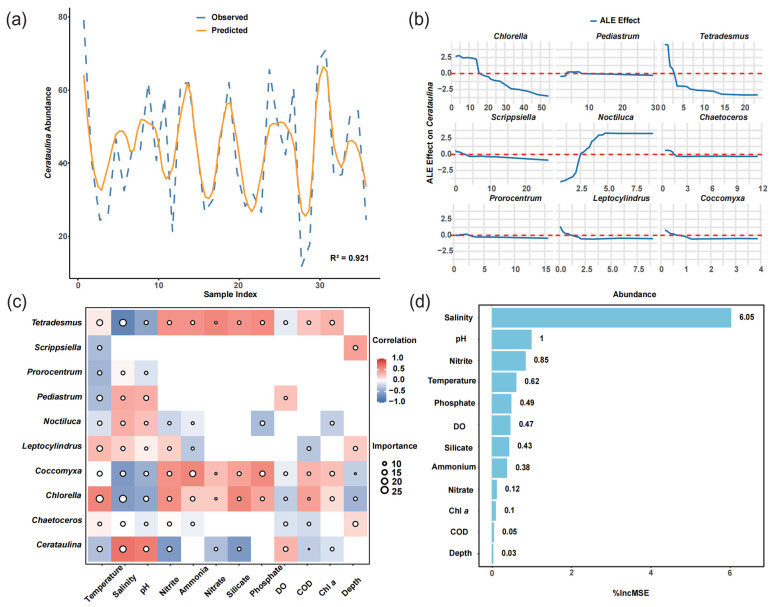
Integrated analysis of RF model performance, interspecific associations, and key species–environment relationships. (**a**) Comparison between predicted (orange solid line) and observed (blue dashed line) species occurrence patterns. (**b**) Accumulated Local Effects (ALE) plots for the top ten species abundances and the dominant species *Cerataulina*. The *x*-axis indicates the abundance of each species, while the *y*-axis shows the average marginal effect of abundance on the predicted value of *Cerataulina*. The horizontal dashed line (ALE = 0) denotes no marginal effect. (**c**) Correlation heatmap between phytoplankton species and environmental variables. The color indicates the strength and direction of Spearman’s correlation coefficient (r). The circle size reflects the %IncMSE importance values derived from the RF model. Blank cells indicate that the correlation or importance is not statistically significant (*p* > 0.05). (**d**) An important ranked list of environmental variables affecting species abundance, based on RF model output.

Our trained random forest model effectively captured the spatiotemporal dynamics of phytoplankton community structure (Table 1 and Figure S1). Despite exhibiting superior performance on the training set, the model maintained a reasonably high R^2^ value on the test set, even with an increase in prediction error. Taking the two dominant species, *Cerataulina* and *Chlorella*, as examples, the model achieved R^2^ values exceeding 98% in-sample and over 82.7% out-of-sample, indicating its satisfactory overall performance in predicting dominant species (Table 1). During cross-validation with varying fold numbers, no obvious overfitting or underfitting was observed. The NRMSE values for both the training and testing sets fluctuated within a reasonable range (approximately 0.1), with an average NRMSE of 0.058 for the testing set. In the testing set, the comparison between the predicted and observed values of *Cerataulina* abundance (Figure 8a) shows that the model effectively captured the abundance trend of the target variable, demonstrating its good generalization ability.

The marginal effects of different species on the abundance of *Cerataulina* within their respective abundance distribution ranges are shown in Figure 8b. Most species exhibit a locally linear negative correlation trend with *Cerataulina*, as demonstrated by the ALE (Accumulated Local Effects) analysis. For example, the ALE curve of *Chlorella* remained above the baseline until an abundance value of 15, indicating a potential positive marginal effect on *Cerataulina* within this abundance range. The curve reached its maximum slope at an abundance value of approximately 15, indicating that *Chlorella* exerts the strongest positive impact on *Cerataulina* around this abundance level. The ALE curves of most species fluctuated slightly around the horizontal dashed line, suggesting that their influence on *Cerataulina* is statistically neutral, which implies that under the ecological conditions during the typhoon period, the impact of these species on *Cerataulina* was relatively mild.

Considering environmental variables, we combined the RF model and Spearman correlation analysis to identify key environmental factors that influence phytoplankton abundance. Our results revealed significant differences in the response patterns of different algal species to various environmental variables (Figure 8c). For the dominant species *Cerataulina*, salinity exhibited both high Importance and high Correlation, indicating that salinity not only makes a significant contribution to the changes in species abundance but also shows a strong positive linear relationship with the abundance of the species. In contrast, while salinity also has a significant impact on the abundance of the species *Chlorella*, this effect may not directly manifest as a negative correlation. There may be non-monotonic or interactive effects, warranting further investigation (high Importance and low Correlation). Silicate shows a strong correlation with *Chlorella* abundance but low predictive power, likely due to collinearity with other variables, suggesting it may be a redundant variable (low Importance and high Correlation). Overall, among the environmental factors related to habitat conditions, temperature, salinity, and pH are the key drivers of variations in phytoplankton abundance. In terms of habitat resource factors, nitrite emerges as an important variable influencing phytoplankton. This result aligns with the key factors identified by the overall RF model (Figure 8d).

## 4. Discussion

### 4.1. Phytoplankton Community Distribution During Typhoon Prapiroon and the Ecological Applications of Macro Barcoding Technology

In this study, *Cerataulina*, a cosmopolitan marine species with frequent bloom reports [24], and *Chlorella*, a genus of Chlorophyta adapted to both aquatic and terrestrial habitats, showed a clear north–south shift along the salinity gradient in Zhanjiang Bay. The distribution of other taxa also matched their known tolerance ranges, confirming salinity as a major determinant of algal biogeography. Low-abundance species were mainly concentrated in the northern low-salinity areas (GD, JS, YG). Notably, our results revealed that Chlorophyta (represented by *Chlorella*) and Bacillariophyta (represented by *Cerataulina*) exhibited an alternating dominance pattern. A similar phenomenon has also been observed in the Beibu Gulf, another region of the South China Sea, where a negative correlation between these two groups was reported [25]. However, this pattern does not necessarily imply direct competition. According to the results of the multi-response random forest model, salinity was identified as the primary driver shaping the overall community structure. Previous mesocosm experiments have demonstrated that salinity stress can inhibit cell division and proliferation in *Chlorella* sp., resulting in lower growth rates compared to low-salinity controls [26,27]. Therefore, the observed distribution pattern may reflect a process of ecological niche partitioning shaped by environmental filtering.

During summer, over 70% of the bay’s water originates from high-salinity oligotrophic seawater of the outer bay, while the Suixi River contributes most nutrients [1]. Hence, even with smaller water inputs, freshwater inflows create favorable niches for nondominant species, maintaining higher diversity under low-salinity conditions. These spatial variations were well-resolved by the 28S rDNA amplicon data.

Typhoon events further intensified the spatial heterogeneity of community structure, particularly between inner estuarine (GD, JS, YG) and outer coastal sites (NS, BG). Uneven disturbance intensities and recovery capacities, coupled with shallow depths and limited water exchange in inner bays, likely enhanced sediment resuspension and freshwater influence. Thus, topography, hydrology, and disturbance gradients jointly shaped the heterogeneous responses of the community.

Zhanjiang Bay’s complex hydrography supports a rich diversity of phytoplankton. Our results captured the dominant taxa previously reported in the region, including *Noctiluca*, *Scrippsiella*, *Nitzschia*, and *Skeletonema* [28,29]. In contrast, species such as *Phaeocystis* observed microscopically in earlier studies were undetected, likely due to the absence of reference sequences in current molecular databases or their minimal abundance in samples.

### 4.2. The Impact of Typhoon Prapiroon on Phytoplankton Community Structure

As one of the most intense meteorological events, typhoons significantly alter the hydrographic and biogeochemical environments of coastal ecosystems. In Zhanjiang Bay, Typhoon Prapiroon brought noticeable changes in physical and chemical conditions. Enhanced wind forcing and heavy rainfall promoted sea-air heat exchange and surface cooling, resulting in decreased temperature, salinity, and pH. These variations are consistent with previous observations of typhoon impacts in other bays and in Zhanjiang Bay itself [1,30,31].

The typhoon also intensified nutrient enrichment via terrestrial runoff. Heavy rainfall increased river discharge and transported nutrient-rich materials from urbanized and industrialized upstream areas [32]. Ammonium and nitrate inputs were further enhanced by the washout of atmospheric ammonium and agricultural fertilizers [12,33,34]. Hydrodynamic disturbance resuspended mangrove sediments, releasing pore-water nutrients such as NH_4_^+^, NO_3_^−^, PO_4_^3−^, and SiO_3_^2−^ through organic matter decomposition and remineralization [35,36]. Consequently, nutrient concentrations increased after the typhoon across all sampling sites, showing the typical decreasing gradient from the inner to the outer bay, as previously reported [37].

Disruptions in nitrogen cycling accompanied changes in nutrient dynamics. Before the typhoon, higher nitrite concentrations relative to nitrate suggested ongoing denitrification or inhibited nitrification. Post-typhoon conditions, characterized by hypoxia and enhanced organic matter remineralization, further promoted denitrification while suppressing the oxidation of NO_2_^−^ to NO_3_^−^. Consequently, NO_2_^−^ accumulation persisted, indicating intensified nitrogen cycling imbalance under typhoon-induced oxygen depletion [34].

Moreover, hydrodynamic alterations driven by the Coriolis effect influenced the movement of water masses in the bay. As Typhoon Prapiroon passed to the left of Zhanjiang Bay, onshore winds generated clockwise circulation, pushing seawater from the outer bay into the upper estuary. This intrusion restricted the outflow of diluted river water and formed a pronounced oceanic front, which, combined with heavy rainfall, limited nutrient dispersion and aggravated hypoxia [38].

Following the disturbance, connectivity between local and regional algal communities declined, reducing the influence of neutral migration processes such as stochastic drift and dispersal. Strengthened environmental filtering and selection pressures became dominant in shaping the community. Overall, Typhoon Prapiroon not only altered the physical and chemical background of Zhanjiang Bay but also reshaped the spatial organization of algal assemblages, driving community reassembly and adaptive shifts in phytoplankton strategies.

### 4.3. Algal Response Patterns

Typhoons modify phytoplankton community structure through both direct and indirect changes in physical and chemical conditions, with species-specific outcomes determined by adaptive strategies [39]. Diatoms, being non-motile, often sink and form resting stages under stratified or quiescent waters [40], but typhoon-induced turbulence can resuspend these resting cells, promoting rapid regrowth and post-disturbance dominance [41]. Owing to their high tolerance to turbulence and rapid growth potential, diatoms often gain a competitive advantage under dynamic hydrological conditions [40]. Small-celled species, in particular, are opportunistic taxa capable of exploiting transient nutrient pulses more efficiently than other phytoplankton [42].

In this study, post-typhoon conditions with reduced temperature and salinity appeared to diminish the competitive edge of *Cerataulina*, resulting in a shift toward smaller *Skeletonema*. This transition coincided with the decline of *Cerataulina* and the rise of *Chaetoceros*, *Bacteriastrum*, and *Ditylum*. Differences in trophic strategies further contributed to transient community instability, consistent with previous reports of typhoon-driven succession from heterotrophy to autotrophy [42].

During the early recovery stage, surface runoff supplied organic detritus that favored heterotrophic and mixotrophic algae such as *Prorocentrum*. As nutrients accumulated, *Cerataulina* and *Prorocentrum* briefly proliferated before the system shifted toward an autotrophic, diatom-dominated assemblage. These results suggest that typhoon-induced nutrient redistribution drives rapid community restructuring. Moreover, the enhanced competitiveness of autotrophic diatoms and the delayed yet synchronous responses of algal and environmental variables indicate that phytoplankton responses weaken or lag over time.

We further infer a shift in nitrogen source preference after the typhoon, with the algal community exhibiting a higher affinity for nitrite than nitrate. Laboratory studies have shown that algae can efficiently assimilate inorganic nitrogen, particularly nitrite and nitrate [43]. Similar observations during monsoon-driven floods indicated that *Cerataulina*, which was dominant before disturbance, favored nitrite [44]. Likewise, *Chlorella* sp. C2 removed up to 60% of nitrite in culture [45]. These findings suggest that enhanced nitrite utilization explains the increased post-typhoon nitrite concentration and divergence from nitrate, with limited effects on community composition.

Overall, algal responses to typhoon-induced disturbances involve integrated strategies encompassing life-history dynamics, trophic succession, and nitrogen source adjustment, collectively driving rapid restructuring and niche reallocation in post-typhoon environments.

### 4.4. Key Factors Driving Phytoplankton Community Structure Changes During Typhoon Prapiroon

Previous analyses based solely on pre- and post-typhoon stages may not fully capture the dynamic responses of environmental variables and phytoplankton communities. To address this limitation, a Random Forest (RF) model was introduced. Unlike traditional linear models, RF—a non-parametric ensemble learning algorithm—effectively captures complex non-linear relationships and variable interactions while maintaining high predictive accuracy [46,47]. Its decision-tree framework automatically evaluates variable importance and remains robust to collinearity, thereby improving the identification of key drivers of community change [48]. Owing to these advantages, machine learning approaches have been increasingly applied in hydrological and ecological research to resolve the non-linear responses of biological communities to environmental disturbances [46,49].

Among the analyzed variables, salinity emerged as the dominant factor shaping species distribution, while nitrite was the most critical nutrient variable. The wide salinity gradient in Zhanjiang Bay (2.51–30.95) supports a diverse assemblage of both freshwater and marine taxa, making salinity a decisive factor in determining species distribution and abundance. Temperature, a major regulator of phytoplankton metabolism and growth [50], showed significant associations with all dominant taxa, reflecting its broad ecological influence. As discussed earlier, resting-stage germination—triggered primarily by light and closely linked to temperature in natural waters [51,52]—may partly explain the observed post-typhoon shifts in community composition. Based on variable importance rankings, parameters related to habitat suitability (temperature, salinity, pH) outweighed nutrient-related factors, suggesting that early post-typhoon recovery was mainly governed by physical constraints, with nutrient effects manifesting later.

Beyond abiotic controls, biotic interactions also contributed to community restructuring. Accumulated Local Effects (ALE) analysis revealed that among the ten most abundant taxa, Noctiluca and *Cerataulina* consistently maintained ALE curves above the baseline with positive slopes, indicating strengthened interspecific facilitation as abundance increased. In contrast, *Chlorella*, *Tetradesmus*, and *Cerataulina* displayed negative linear correlations at low abundance levels. Similarly to our findings, Li et al. [53] reported synchronous increases in *Noctiluca* and diatoms after typhoon events, attributed to trophic coupling—diatom blooms likely providing food resources for heterotrophic *Noctiluca*.

Overall, typhoon-induced salinity reduction from freshwater inflow was the primary external driver of phytoplankton community shifts, while temperature served as the most pervasive regulatory factor. Meanwhile, interspecific interactions further modulated the reassembly process, collectively shaping the complex post-disturbance response of algal communities in Zhanjiang Bay. While our study provides insights into phytoplankton dynamics, it has certain limitations. Specifically, it focused on the eukaryotic fraction, excluding prokaryotic primary producers such as cyanobacteria. Consequently, interpretations pertain primarily to eukaryotes, and future studies including prokaryotes would help better understand overall phytoplankton community dynamics.

## 5. Conclusions

Our study demonstrates the value of metabarcoding analysis in investigating the composition of the phytoplankton community in Zhanjiang Bay and the changes in community structure under the influence of Typhoon Prapiroon. Multivariate analysis revealed that the typhoon altered the ecological environment of Zhanjiang Bay. PCA, PCoA, and NMDS showed that the typhoon exacerbated the existing differences in geographical distribution patterns, with significant spatial heterogeneity of algae in the estuary and outer bay. Association network analysis revealed that the pre-existing competition or resource partitioning relationships among populations were weakened by the disturbance, and the community’s overall response to environmental factors became more subdued and delayed. The NCM analysis indicated that non-neutral assembly mechanisms, such as environmental filtering and biotic interactions, played a role in community assembly following the typhoon disturbance. RF results indicated that salinity was the key determinant shaping algal community structure during the Typhoon event, with the community reconstruction process primarily influenced by habitat suitability factors. Additionally, RF effectively identified and revealed the complex ecological relationships among both biotic and abiotic factors. Our study provides a foundation for understanding the composition and dynamic changes in coastal phytoplankton communities during typhoon events, thereby enhancing our understanding of algal community ecological response patterns under extreme environmental conditions. The next challenge is to further expand the metabarcoding analysis to tropical cyclone events of varying intensities, paths, and rainfall conditions, incorporating additional environmental factors and microbial variables, to establish a microbial ecological baseline framework with environmental indicator significance.

## Figures and Tables

**Table 1:** Performance metrics of the random forest (RF) model in predicting the abundance of *Cerataulina* and *Chlorella*.

Model Performance Metrics	Predicting *Cerataulina*		Predicting *Chlorella*	
	Training (70%)	Test (30%)	Training (70%)	Test (30%)
Root mean square error (RMSE)	2.28	4.81	2.27	6.02
Mean absolute error (MAE)	1.8	3.59	1.75	4.58
Coefficient of determination (R^2^)	0.98	0.92	0.98	0.82

## Data Availability

The original contributions presented in this study are included in the article or Supplementary Material. Further inquiries can be directed to the corresponding author.

## Supplementary Materials

The following supporting information can be downloaded at: https://www.mdpi.com/article/10.3390/microorganisms13112609/s1, Figure S1: Assessment of model generalization and stability. The normalized root mean square error (NRMSE) was calculated for each fold of 10-fold cross-validation on the test set. The x-axis represents the fold number (1–10) in the cross-validation, while the y-axis shows the corresponding NRMSE values, reflecting the variation in prediction error across different data partitions; Figure S2: The Shannon-Wiener index reflects microbial diversity within each sample. A diversity curve was constructed based on sequencing depth and microbial diversity indices across samples. When the curve becomes flat, it indicates that the sequencing depth is sufficient to capture the majority of microbial diversity within the sample; Figure S3: Temporal trends of environmental parameters at different stages. Variations in (a) temperature, (b) pH, (c) COD, (d) DO, (e) nitrate, (f) ammonium, (g) silicate, and (h) phosphate across different sampling sites during Typhoon Prapiroon.

## References

[1] Chen C., Lao Q., Zhou X., Jin G., Zhu Q., Chen F. (2024). Tracks of typhoon movement (left and right sides) control marine dynamics and eco-environment in the coastal bays after typhoons: A case study in Zhanjiang Bay. Sci. Total Environ..

[2] Wang Z., Xia N., Zhao X., Ji X., Wang J. (2024). Comprehensive risk assessment of typhoon disasters in China’s coastal areas based on multi-source geographic big data. Sci. Total Environ..

[3] Park D.S.R., Ho C.H., Kim J.H. (2014). Growing threat of intense tropical cyclones to East Asia over the period 1977–2010. Environ. Res. Lett..

[4] Srinivas H., Nakagawa Y. (2008). Environmental implications for disaster preparedness: Lessons learnt from the Indian Ocean Tsunami. J. Environ. Manag..

[5] Pandey R.S., Liou Y.A. (2022). Typhoon strength rising in the past four decades. Weather Clim. Extremes.

[6] Wang J., Zhu S., Liu J., Wang X., Wang J., Xu J., Yang Y. (2023). Frequency, intensity and influences of tropical cyclones in the Northwest Pacific and China, 1977–2018. Sustainability.

[7] Gobler C.J., Doherty O.M., Hattenrath-Lehmann T.K., Griffith A.W., Kang Y., Litaker R.W. (2017). Ocean warming since 1982 has expanded the niche of toxic algal blooms in the North Atlantic and North Pacific oceans. Proc. Natl. Acad. Sci. USA.

[8] Grattan L.M., Holobaugh S., Morris J.G. (2016). Harmful algal blooms and public health. Harmful Algae.

[9] Wang T., Zhang S., Chen F., Ma Y., Jiang C., Yu J. (2021). Influence of sequential tropical cyclones on phytoplankton blooms in the northwestern South China Sea. J. Oceanol. Limnol..

[10] Thompson P.A., Paerl H.W., Campbell L., Yin K., McDonald K.S. (2023). Tropical cyclones: What are their impacts on phytoplankton ecology?. J. Plankton Res..

[11] Behrenfeld M.J., Randerson J.T., McClain C.R., Feldman G.C., Los S.O., Tucker C.J., Falkowski P.G., Field C.B., Frouin R., Esaias W.E. (2001). Biospheric primary production during an ENSO transition. Science.

[12] Zhang P., Long H., Li Z., Chen R., Peng D., Zhang J. (2024). Effects of typhoon events on coastal hydrology, nutrients, and algal bloom dynamics: Insights from continuous observation and machine learning in semi-enclosed Zhanjiang Bay, China. Sci. Total Environ..

[13] Wu X.Y., Yang Y.F. (2011). Heavy metal (Pb, Co, Cd, Cr, Cu, Fe, Mn and Zn) concentrations in harvest-size white shrimp *Litopenaeus vannamei* tissues from aquaculture and wild source. J. Food Compos. Anal..

[14] Wang G., Zhang Y., Guan D., Xiao L., Singh M. (2021). The potential of mature *Sonneratia apetala* plantations to enhance carbon stocks in the Zhanjiang Mangrove National Nature Reserve. Ecol. Indic..

[15] Chen F., Huang C., Lao Q., Zhang S., Chen C., Zhou X., Lu X., Zhu Q. (2021). Typhoon control of precipitation dual isotopes in southern China and its palaeoenvironmental implications. J. Geophys. Res. Atmos..

[16] Adhikari M.D., Park S., Yum S.G. (2025). Coastal vulnerability to extreme weather events: An integrated analysis of erosion, sediment movement, and land subsidence based on multi-temporal optical and SAR satellite data. J. Environ. Manag..

[17] Chai Z.Y., He Z.L., Deng Y.Y., Yang Y.F., Tang Y.Z. (2018). Cultivation of seaweed *Gracilaria lemaneiformis* enhanced biodiversity in a eukaryotic plankton community as revealed via metagenomic analyses. Mol. Ecol..

[18] Bolger A.M., Lohse M., Usadel B. (2014). Trimmomatic: A flexible trimmer for Illumina sequence data. Bioinformatics.

[19] Edgar R.C. (2010). Search and clustering orders of magnitude faster than BLAST. Bioinformatics.

[20] (2007). The Specification for Marine Monitoring—Part 4: Seawater Analysis.

[21] Harley J.R., Lanphier K., Kennedy E., Whitehead C., Bidlack A. (2020). Random forest classification to determine environmental drivers and forecast paralytic shellfish toxins in Southeast Alaska with high temporal resolution. Harmful Algae.

[22] Liu M., Huang Y., Hu J., He J., Xiao X. (2023). Algal community structure prediction by machine learning. Environ. Sci. Ecotechnol..

[23] Breiman L. (2001). Random forests. Mach. Learn..

[24] Ianora A., Casotti R., Bastianini M., Brunet C., d’Ippolito G., Acri F., Fontana A., Cutignano A., Turner J.T., Miralto A. (2008). Low reproductive success for copepods during a bloom of the non-aldehyde-producing diatom *Cerataulina pelagica* in the North Adriatic Sea. Mar. Ecol..

[25] Peng C., Qin Y., Liu Y., Sun D., Xie Z., Jia J., Li H., Liu X., Cao H., Gong B. (2025). Environmental Factors, Not Biotic Competitive Interactions, Drive the Relative Abundance of Diatoms and Chlorophyta in the Coastal Areas of the Beibu Gulf: Evidence From 18S rDNA Metabarcoding and Partial Least Squares-Path Modeling Analysis. Ecol. Evol..

[26] Li X., Yuan Y., Cheng D., Gao J., Kong L., Zhao Q., Wei W., Sun Y. (2018). Exploring stress tolerance mechanism of evolved freshwater strain *Chlorella* sp. S30 under 30 g/L salt. Bioresour. Technol..

[27] Wang T., Li D., Tian X., Huang G., He M., Wang C., Kumbhar A.N., Woldemicael A.G. (2024). Mitigating salinity stress through interactions between microalgae and different forms (free-living & alginate gel-encapsulated) of bacteria isolated from estuarine environments. Sci. Total Environ..

[28] Zhang J., Fu M., Zhang P., Sun D., Peng D. (2023). Unravelling nutrients and carbon interactions in an urban coastal water during algal bloom period in Zhanjiang Bay, China. Water.

[29] Li L., Lü S., Cen J. (2019). Spatio-temporal variations of Harmful algal blooms along the coast of Guangdong, Southern China during 1980–2016. J. Oceanol. Limnol..

[30] Subrahmanyam M.V. (2015). Impact of typhoon on the north-west Pacific sea surface temperature: A case study of Typhoon Kaemi (2006). Nat. Hazards.

[31] Jiang T., Wu G., Niu P., Cui Z., Bian X., Xie Y., Shi H., Xu X., Qu K. (2022). Short-term changes in algal blooms and phytoplankton community after the passage of Super Typhoon Lekima in a temperate and inner sea (Bohai Sea) in China. Ecotoxicol. Environ. Saf..

[32] Zhang J., Zhang Y., Zhang P., Li Y., Li J., Luo X., Xu J., Zhao L. (2021). Seasonal phosphorus variation in coastal water affected by the land-based sources input in the eutrophic Zhanjiang Bay, China. Estuar. Coast. Shelf Sci..

[33] Chen F., Lao Q., Liu M., Huang P., Chen B., Zhou X., Chen P., Chen K., Song Z., Cai M. (2022). Impact of intensive mariculture activities on microplastic pollution in a typical semi-enclosed bay: Zhanjiang Bay. Mar. Pollut. Bull..

[34] Herbeck L.S., Unger D., Krumme U., Liu S.M., Jennerjahn T.C. (2011). Typhoon-induced precipitation impact on nutrient and suspended matter dynamics of a tropical estuary affected by human activities in Hainan, China. Estuar. Coast. Shelf Sci..

[35] Taillardat P., Ziegler A.D., Friess D.A., Widory D., David F., Ohte N., Nakamura T., Evaristo J., Thanh-Nho N., Van Vinh T. (2019). Assessing nutrient dynamics in mangrove porewater and adjacent tidal creek using nitrate dual-stable isotopes: A new approach to challenge the Outwelling Hypothesis?. Mar. Chem..

[36] Wang F., Cheng P., Chen N., Kuo Y.M. (2021). Tidal driven nutrient exchange between mangroves and estuary reveals a dynamic source-sink pattern. Chemosphere.

[37] Lao Q., Wu J., Chen F., Zhou X., Li Z., Chen C., Zhu Q., Deng Z., Li J. (2022). Increasing intrusion of high salinity water alters the mariculture activities in Zhanjiang Bay during the past two decades identified by dual water isotopes. J. Environ. Manag..

[38] Zhao Y., Uthaipan K., Lu Z., Li Y., Liu J., Liu H., Gan J., Meng F., Dai M. (2021). Destruction and reinstatement of coastal hypoxia in the South China Sea off the Pearl River estuary. Biogeosciences.

[39] Liu J., Long X., Bai M., Liu Y., Zhang Y., Jia W. (2025). Climate-adaptive flocculant design: Mechanistic advances and operational challenges for harmful algal blooms control. J. Environ. Manag..

[40] Lim Y.K., Hong S., Baek S.H. (2022). Potential influence of the proliferation of sediment-based diatoms on blooms of a harmful dinoflagellate *Cochlodinium polykrikoides*: A microcosm approach. J. Appl. Phycol..

[41] Baek S.H., Shimode S., Shin K., Han M.S., Kikuchi T. (2009). Growth of dinoflagellates, *Ceratium furca* and *Ceratium fusus* in Sagami Bay, Japan: The role of vertical migration and cell division. Harmful Algae.

[42] Tsuchiya K., Kuwahara V.S., Yoshiki T.M., Nakajima R., Shimode S., Kikuchi T., Toda T. (2017). Response of phytoplankton and enhanced biogeochemical activity to an episodic typhoon event in the coastal waters of Japan. Estuar. Coast. Shelf Sci..

[43] Taziki M., Ahmadzadeh H., Murry M.A., Lyon S.R. (2015). Nitrate and nitrite removal from wastewater using algae. Curr. Biotechnol..

[44] Vineetha G., Kripa V., Karati K.K., Rehitha T.V., Vishal C.R., Vineetha V., Manu M. (2020). Impact of a catastrophic flood on the heavy metal pollution status and the concurrent responses of the bentho-pelagic community in a tropical monsoonal estuary. Mar. Pollut. Bull..

[45] Zhang X., Chen H., Chen W., Qiao Y., He C., Wang Q. (2014). Evaluation of an oil-producing green alga *Chlorella* sp. C2 for biological DeNOx of industrial flue gases. Environ. Sci. Technol..

[46] Huang Y., Chen S., Tang X., Sun C., Zhang Z., Huang J. (2024). Dynamic patterns and potential drivers of river water quality in a coastal city: Insights from a machine-learning-based framework and water management. J. Environ. Manag..

[47] Liao Z., Yu K., Chen B., Huang X., Qin Z., Yu X. (2021). Spatial distribution of benthic algae in the South China Sea: Responses to gradually changing environmental factors and ecological impacts on coral communities. Divers. Distrib..

[48] Cheng Y., Bhoot V.N., Kumbier K., Sison-Mangus M.P., Brown J.B., Kudela R., Newcomer M.E. (2021). A novel random forest approach to revealing interactions and controls on chlorophyll concentration and bacterial communities during coastal phytoplankton blooms. Sci. Rep..

[49] Chen J., Huang G., Chen W. (2021). Towards better flood risk management: Assessing flood risk and investigating the potential mechanism based on machine learning models. J. Environ. Manag..

[50] Sunagawa S., Coelho L.P., Chaffron S., Kultima J.R., Labadie K., Salazar G., Djahanschiri B., Zeller G., Mende D.R., Alberti A. (2015). Structure and function of the global ocean microbiome. Science.

[51] Liu S., Cui Z., Zhao Y., Chen N. (2022). Composition and spatial-temporal dynamics of phytoplankton community shaped by environmental selection and interactions in the Jiaozhou Bay. Water Res..

[52] Tsai S.F., Wu L.Y., Chou W.C., Chiang K.P. (2018). The dynamics of a dominant dinoflagellate, Noctiluca scintillans, in the subtropical coastal waters of the Matsu archipelago. Mar. Pollut. Bull..

[53] Li H., Li S., Zhang M., Li X., Xu Z., Ma H., Liang S., Song D., Li J., Ma Y. (2024). Typhoon-induced stormwater drives nutrient dynamics and triggers phytoplankton blooms in Laizhou Bay, China. Mar. Environ. Res..

