# Supplementary material for: Changes in Eukaryotic Phytoplankton Community Structure Induced by a Typhoon Event: A Case Study in Zhanjiang Bay, China
Source: Microorganisms. 2025 Nov 16;13(11):2609. doi: 10.3390/microorganisms13112609 (PMC12654317; doi:10.3390/microorganisms13112609)
Supplement: Supplementary file 1 [file microorganisms-13-02609-s001.zip › microorganisms-3969907-supplementary.pdf]

Supplementary Materials:

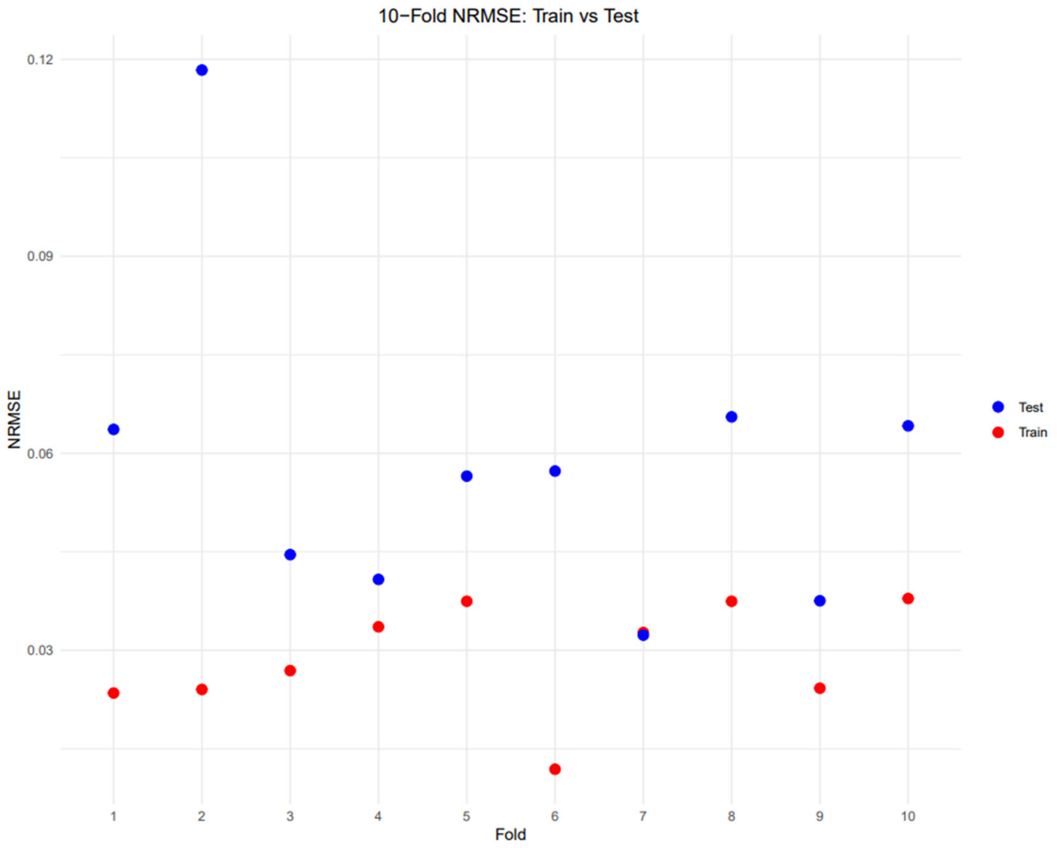

Figure S1. Assessment of model generalization and stability. The normalized root mean square error (NRMSE) was calculated for each fold of 10-fold cross-validation on the test set. The x-axis represents the fold number (1–10) in the cross-validation, while the y-axis shows the corresponding NRMSE values, reflecting the variation in prediction error across different data partitions.

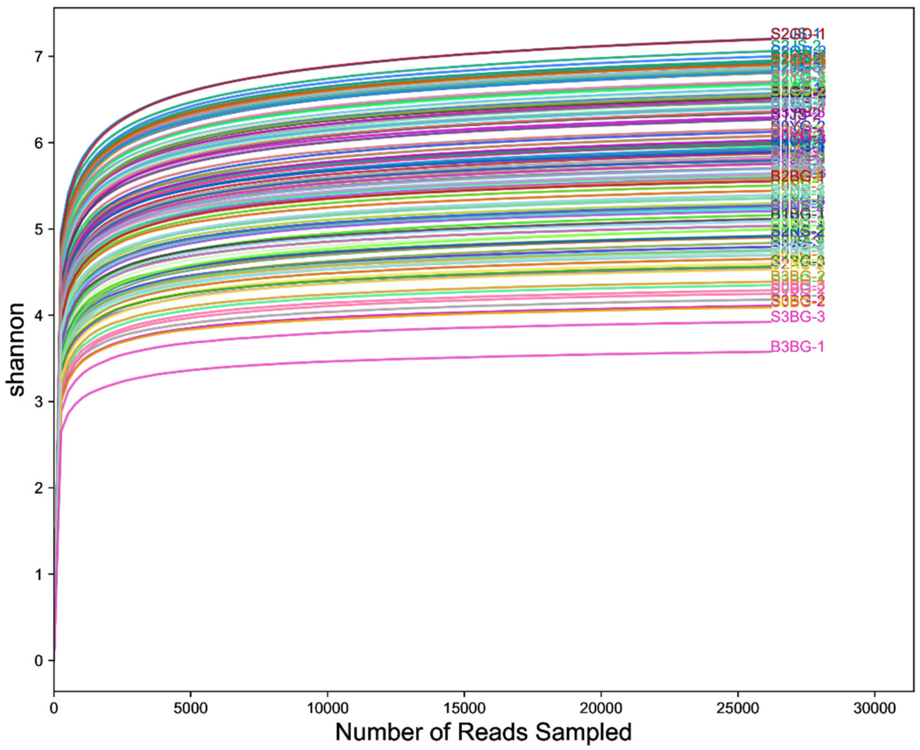

Figure S2. The Shannon-Wiener index reflects microbial diversity within each sample. A diversity curve was constructed based on sequencing depth and microbial diversity indices across samples. When the curve becomes flat, it indicates that the sequencing depth is sufficient to capture the majority of microbial diversity within the sample.

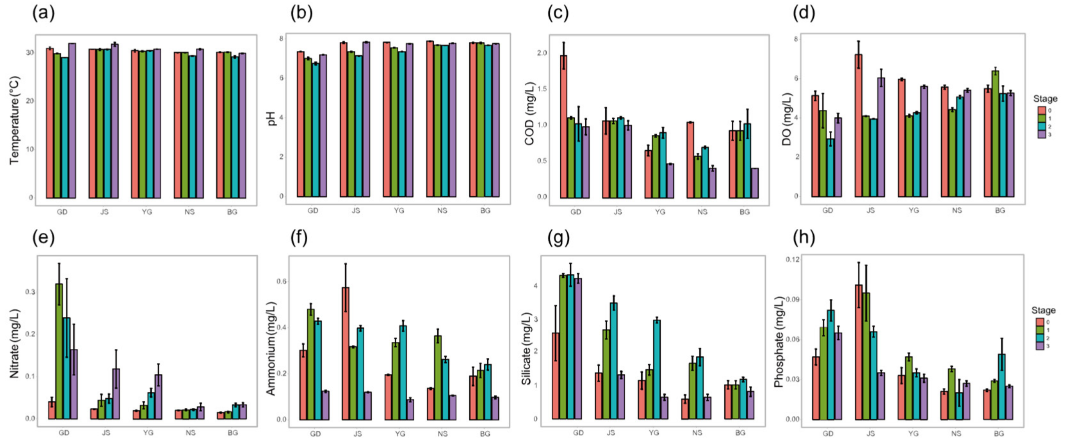

Figure S3. Temporal trends of environmental parameters at different stages. Variations in (a) temperature, (b) pH, (c) COD, (d) DO, (e) nitrate, (f) ammonium, (g) silicate, and (h) phosphate across different sampling sites during Typhoon Prapiroon.
